# Complete Genome Sequence of Rhodococcus qingshengii Strain VKM Ac-2784D, Isolated from Elytrigia repens Rhizosphere

**DOI:** 10.1128/MRA.00107-21

**Published:** 2021-03-18

**Authors:** Ivan S. Petrushin, Yulia A. Markova, Marina S. Karepova, Yulia V. Zaytseva, Lyudmila A. Belovezhets

**Affiliations:** a Siberian Institute of Plant Physiology and Biochemistry, Siberian Branch of the Russian Academy of Sciences, Irkutsk, Russia; b Irkutsk State University, Irkutsk, Russia; c Yaroslavl State University, Yaroslavl, Russia; d A. E. Favorsky Irkutsk Institute of Chemistry, Siberian Branch of the Russian Academy of Sciences, Irkutsk, Russia; University of Arizona

## Abstract

Rhizosphere bacteria are considered to be promising destructors of oil and its components. Bacterial species of the genus *Rhodococcus* can degrade a variety of hydrocarbons and are widely used for the bioremediation of polluted environments. Here, we report the complete genome sequence of Rhodococcus qingshengii strain VKM Ac-2784D.

## ANNOUNCEMENT

Products of petroleum origin (oil and its components) are dangerous pollutants of the soil. Bacterial species of the genus *Rhodococcus* have the ability to degrade a variety of hydrocarbons in contaminated soil and are useful for the bioremediation of polluted environments (1–3). We isolated Rhodococcus qingshengii strain VKM Ac-2784D from the rhizosphere of couch grass (Elytrigia repens) growing at an oil-polluted site near Zalary, Irkutsk State, Russia (53°40′15″N, 102°18′29″E) (4, 5). This strain can effectively degrade oil and some model compounds (naphthalene, anthracene, and phenanthrene) (5, 6). Such activity decreases the negative effect of soil pollution and allows plants to resume growth processes (7). We suggest that the bioremediation activity of the strain in this study is associated with the production of biosurfactants and phytohormones (5).

We isolated bacteria from soil near the roots as described previously (4). To isolate bacteria, 10 g of soil near the roots was put into 100 ml sterile water and shaken for 15 min. The resulting suspension was inoculated into an accumulative medium of the following composition (g/liter): KNO_3_, 4.0; KH_2_PO_4_, 0.6; Na_2_HPO_4_, 12; H_2_O, 1.4; MgSO_4_, 7; H_2_O, 0.8. The medium was supplemented with only the following carbon sources: hexane, motor oil, waste motor oil, and gasoline. The strains were grown on a shaker for 30 days at 26°C. To obtain a pure culture of hydrocarbon-oxidizing bacteria, we used a solid agar medium.

DNA was extracted from the obtained bacterial strain after bead beating using the DNeasy PowerLyzer microbial kit (Qiagen, USA) according to the manufacturer’s protocols. A sequence library was generated from DNA using the HyperPlus DNA sample preparation kit (KAPA Biosystems, USA). The universal bacterial primers 518F and 1064R were used to amplify the V4 to V6 hypervariable region of the bacterial 16S rRNA gene. Strain *R. qingshengii* VKM Ac-2784D showed the highest 16S rRNA gene phylogenetic affiliation to species belonging to the family *Nocardiaceae*.

Whole-genome sequencing was performed using the Illumina NextSeq platform with paired-end chemistry (2 × 100 bp). A total of 9,362,538 paired-end reads was obtained, giving a coverage depth of 360×. Raw read error correction and filtering was performed using the fastp tool with default settings (8). A draft assembly was built using SPAdes version 3.14.0 (9) with default settings and the “isolate” option enabled. This draft assembly contained 105 contigs with an *N*_50_ value of 961,557 bp and a largest contig length of 1,207,723 bp.

The resulting contigs were combined into a whole chromosome using Ragout version 2.3 with default settings (10) (https://github.com/fenderglass/Ragout) using the Rhodococcus erythropolis SK121 whole-genome sequencing assembly (GenBank accession no. ACNO00000000) and the Rhodococcus erythropolis CCM2595 chromosome (NC_022115) as the reference genome sequences. The final assembly contained 1 chromosome, with a total genome sequence size of 6,251,765 bp and a GC content of 62.5%.

Genome completeness analysis with BUSCO version 5.0.0 using the data set “actinobacteria_phylum_odb10” with 356 benchmarking universal single-copy orthologs (BUSCOs) and default settings (11) gave results of 99.4% complete and single-copy, 0.6% duplicated, and no missing BUSCOs. The genome sequence was then annotated using the NCBI Prokaryotic Genome Annotation Pipeline (PGAP) (12). This assembly contained 5,775 genes encoding 5,716 protein-coding sequences, 53 tRNAs, 3 noncoding RNAs (ncRNAs), 3 rRNAs, and 49 pseudogenes, as identified by PGAP.

**FIG 1:**
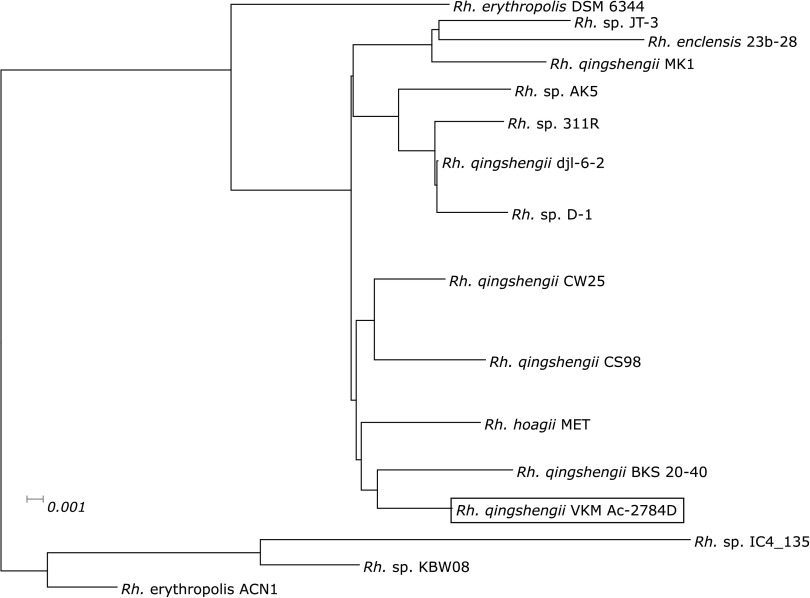
Phylogenetic tree of related *Rhodococcus* species.

To compare the genomic features of Rhodococcus qingshengii strain VKM Ac-2784D with related species, we built a phylogenetic tree for some of these species (Fig. 1). First, we built a complete tree for >390 strains of *Rhodococcus* available in GenBank, and then we chose the 16 closest to them for a detailed tree. The tree is based on ∼400 universal marker genes determined by PhyloPhlAn version 3.0 (13) (with default settings using the maximum-likelihood method).

We performed a search for biosynthetic gene clusters (BGCs) encoding secondary metabolites with the Web version of antiSMASH version 6.0 (14) and found 19 functional clusters (regions) on the chromosome, including clusters for the production of heterobactin, ectoine, erythrochelin, bacteriocin, ectoine, and heterobactin A/heterobactin S2 (nonribosomal peptide synthetase [NRPS]).

Availability of the Rhodococcus qingshengii strain VKM Ac-2784D genome sequence will aid in understanding its hydrocarbon-degrading mechanisms and broaden our knowledge of plant-bacteria interactions.

### Data availability.

This whole-genome shotgun sequence has been deposited at DDBJ/ENA/GenBank under the accession no. CP064920. The raw reads are available via BioProject accession no. PRJNA673759 or SRR12964023. The version described in this paper is the first version.
